# Supplementary material for: Identification of Divergent Isolates of Banana Mild Mosaic Virus and Development of a New Diagnostic Primer to Improve Detection
Source: Pathogens. 2020 Dec 12;9(12):1045. doi: 10.3390/pathogens9121045 (PMC7764570; doi:10.3390/pathogens9121045)
Supplement: Supplementary file 1 [file pathogens-09-01045-s001.zip › Suppl file S2-molecular test-BanMMV-FV.docx]

**Molecular diagnostic test used to detect BanMMV in this study: IC- RT-PCR**

Twenty-five microlitres of an antibody mixture containing 5 μg/ml of BanMMV IgG (J. Thomas, unpublished) in sterile carbonate coating buffer was added to each thin-walled PCR tube and incubated for 2 h at 37°C before being washed three times for three min with PBS-T (0.05 M phosphate buffer pH 7.4, 0.15 M NaCl, 2.7 mM KCl, 0.05% Tween20). Twenty-five microlitres of the clarified leaf extract (crude extract) was then added to the washed, coated tube and incubated at room temperature for 4°C overnight. Tubes were washed three times for three min at room temperature with PBS-T and rinsed once with sterile distilled water.

After the water was removed from the immunocapture tubes, reverse transcription was carried out in order to generate cDNA for the virus with RNA genomes. Diluted primers (750 nM Poty1 and 750 nM CMV3′; 12.5 μl total) were added to the immunocapture tube, which was incubated at 80ºC for 10 min, then rapidly chilled on ice. The following reagents were added (final concentration in 7.5 μl): 1× first strand buffer (Invitrogen, USA), 10 mM DTT, 500 nM dNTPs, 10 U RNaseOUT (Invitrogen), 50 U Superscript III reverse transcriptase (Invitrogen), and the tubes incubated at 50 ºC for 45 min, then 70°C for 15 min.

Two microliters of the cDNA mixture was added to 23 μl of the BanMMV PCR mastermix, prepared as follows: 1× MangoTaq coloured PCR buffer (Bioline, Australia), 2 mM MgCl_2_, 200 nM dNTPs, 400 nM Poty1 primer (Table 1), 800 nM BanMMCP2 primer, 2.5 U MangoTaq DNA polymerase (Bioline, Australia). The thermal cycling conditions were: 94°C for 1 min, followed by 35 cycles of 94°C for 20 s, 60°C for 20 s and 72°C for 20 s, then 72°C for 3 min. The expected product size was ~280 bp.

In all cases, the PCR products were separated by electrophoresis in a 1% agarose gel in 0.5× TBE, and stained with GelRed (Biotium).
