# Supplementary material for: Identification of Divergent Isolates of Banana Mild Mosaic Virus and Development of a New Diagnostic Primer to Improve Detection
Source: Pathogens. 2020 Dec 12;9(12):1045. doi: 10.3390/pathogens9121045 (PMC7764570; doi:10.3390/pathogens9121045)
Supplement: Supplementary file 1 [file pathogens-09-01045-s001.zip › Suppl file S3- ISEM procedure-FV.docx]

**Immunosorbent Electron Microscopy (ISEM) of Viral Minipreps:**

Three to five clonal plants of each germplasm line were grown out from tissue culture under glasshouse conditions. Plants were sampled at three and six months after deflasking. Samples at each time point were pooled for the indexing process.

For the viral miniprep, the 2 g subsample of leaf lamina and midrib tissue was ground in 6 mL of extraction buffer (0.2 M potassium phosphate buffer (pH 7.0), 15 mM EDTA, 2% polyvinylpyrrolidone, 2% polyethylene glycol and 0.4% Na_2_SO_3_) using a mortar and pestle, and the slurry filtered through cheesecloth. The filtrate was clarified by centrifugation at 12,000 *g* for 15 min at 5°C and the supernatant recovered. Triton X-100 (120 µL of 33% v/v solution) was added and the mix was vortexed for 1 min. A cushion of 1 mL of 30% sucrose in 0.2M potassium phosphate buffer at pH 7.0 was under laid and the tube ultracentrifuged at 163,000 ***g*** for 30 min at 5°C. This pellet was resuspended in 100 µL of 10 mM potassium phosphate buffer, pH 7.0 and transferred to a microcentrifuge tube. Thirty µL of chloroform was added to the tube and the mixture vortexed for 30 s prior to centrifugation for 5 min at 12,000 ***g***. The aqueous supernatant was stored at -70°C.

ISEM was conducted as follows. Nitrocellulose- or Nicolodion-coated, carbon-stabilised copper electron microscope grids were placed film side down on 10 µl droplets of antibody mixture containing 5 µg mL^-1^ each of SCBV IgGs (Agdia, USA), BSMYV IgG (J. Vo and A. Geering, unpublished), BBrMV IgG (Agdia, USA) and BanMMV IgG (J. Thomas, unpublished), and 0.2 µg mL^-1^ (a 1/200 dilution) of  CMV IgG ( Sediag, France).

Grids were incubated in a humid chamber for 30–60 minutes at room temperature, then washed with 20 drops of a 10 mM sodium phosphate buffer, pH 7.0 and dried with filter paper. The grids were placed film side down on a 10 µL drop of the viral miniprep and incubated in a humid chamber at 4°C overnight. The grids were then washed again with 20 drops of 10 mM sodium phosphate buffer, pH 7.0 and dried. The grids were negatively contrasted with five drops of 1% ammonium molybdate, pH 6.8–7.0 and touch dried with filter paper. Grids were observed for 10 minutes with an electron microscope (Hitachi H-7000 or JEOL-TEM-1400). Micrographs were captured on film or using an Orius digital camera (Gatan Inc, USA).
