# Supplementary material for: Identification of Divergent Isolates of Banana Mild Mosaic Virus and Development of a New Diagnostic Primer to Improve Detection
Source: Pathogens. 2020 Dec 12;9(12):1045. doi: 10.3390/pathogens9121045 (PMC7764570; doi:10.3390/pathogens9121045)
Supplement: Supplementary file 1 [file pathogens-09-01045-s001.zip › Suppl file S6- SNPs-FV.docx]

**Table1**. Distrubution of SNPs on MT872724

| Isolate | Position des SNPs | Change | %SNP | Region | Region delimitation | Coverage |
| --- | --- | --- | --- | --- | --- | --- |
| Isolate1 | 421 | T🡪C | 6.1 | RdRp | 64 🡪5391 | 197 |
|  | 438 | G🡪A | 7 | RdRp | 64 🡪5391 | 214 |
|  | 738 | T🡪C | 1.5 | RdRp | 64 🡪5391 | 337 |
|  | 1,448 | G🡪T | 8.2 | RdRp | 64 🡪5391 | 268 |
|  | 1,692 | T🡪C | 4.9 | RdRp | 64 🡪5391 | 184 |
|  | 1,697 | T🡪A | 2.7 | RdRp | 64 🡪5391 | 184 |
|  | 1,715 | T🡪C | 2.3 | RdRp | 64 🡪5391 | 173 |
|  | 1,821 | G🡪A | 4.1 | RdRp | 64 🡪5391 | 123 |
|  | 1,881 | G🡪A | 22.7 | RdRp | 64 🡪5391 | 132 |
|  | 2,628 | C🡪T | 1.6 | RdRp | 64 🡪5391 | 183 |
|  | 2,985 | C🡪T | 3.2 | RdRp | 64 🡪5391 | 190 |
|  | 3,027 | T🡪C | 2.6 | RdRp | 64 🡪5391 | 190 |
|  | 3,031 | A🡪G | 2.6 | RdRp | 64 🡪5391 | 193 |
|  | 3,058 | T🡪C | 2.3 | RdRp | 64 🡪5391 | 177 |
|  | 3,119 | G🡪A | 2.3 | RdRp | 64 🡪5391 | 175 |
|  | 3,587 | G🡪A | 4.6 | RdRp | 64 🡪5391 | 130 |
|  | 3,843 | G🡪A | 17.8 | RdRp | 64 🡪5391 | 152 |
|  | 3,852 | G🡪A | 3.5 | RdRp | 64 🡪5391 | 144 |
|  | 5,211 | C🡪T | 9.1 | RdRp | 64 🡪5391 | 66 |
|  | 5,289 | A🡪G | 9.1 | RdRp | 64 🡪5391 | 55 |
|  | 5,446 | C🡪T | 7.7 | TGB protein 2 | 5354🡪 6028 | 91 |
|  | 5,567 | G🡪A | 4.3 | TGB protein 2 | 5354🡪 6028 | 93 |
|  | 5,944 | T🡪C | 3.3 | TGB protein 2 | 5354🡪 6028 | 123 |
|  | 6,110 | T🡪A | 4.6 | TGB protein 3 | 6028🡪 6366 | 152 |
|  | 6,427 | T🡪C | 9.1 | TGB protein 4 | 6281🡪 6493 | 55 |
|  | 6,488 | C🡪A | 8.9 | TGB protein 4 | 6281🡪 6493 | 56 |
|  | 6,812 | A🡪G | 8.7 | CP | 6564🡪 7241 | 46 |
|  | 7,239 | T🡪G | 94.1 | CP | 6564🡪 7241 | 17 |
|  | 7,241 | A🡪T | 94.1 | CP | 6564🡪 7241 | 17 |
|  | 7,247 | T🡪G | 92.9 | NCR | NCR | 14 |
|  | 7,255 | G🡪T | 66.7 | NCR | NCR | 3 |

**Table2.** Distribution of SNPs on MT872725

| Isolate | Position des SNPs | Change | %SNP | Region | Region delimitation | Coverage |
| --- | --- | --- | --- | --- | --- | --- |
| Isolate2 | 1808 | G🡪A | 1.5 | RdRp | 50 🡪5356 | 409 |
|  | 2138 | C🡪T | 1.7 | RdRp | 50 🡪5356 | 291 |
|  | 2564 | A🡪G | 2 | RdRp | 50 🡪5356 | 295 |
|  | 2603 | G🡪A | 1.7 | RdRp | 50 🡪5356 | 294 |
|  | 2955 | A🡪G | 1.2 | RdRp | 50 🡪5356 | 322 |
|  | 3811 | G🡪A | 3.6 | RdRp | 50 🡪5356 | 197 |
|  | 4023 | T🡪C | 2.5 | RdRp | 50 🡪5356 | 239 |
|  | 5113 | G🡪A | 2.6 | RdRp | 50 🡪5356 | 227 |
|  | 5170 | C🡪T | 5.5 | RdRp | 50 🡪5356 | 201 |
|  | 5827 | C🡪T | 3 | TGB2 | 5319🡪5993 | 232 |
|  | 6062 | G🡪T | 2 | TGB3 | 5993🡪6331 | 254 |
|  | 6192 | G🡪A | 2.7 | TGB3 | 5993🡪6331 | 149 |
|  | 6330 | T🡪C | 4 | TGB3 | 5993🡪6331 | 200 |
|  | 6879 | A🡪G | 5.2 | CP | 6528🡪7244 | 115 |
