# Supplementary material for: Identification of Divergent Isolates of Banana Mild Mosaic Virus and Development of a New Diagnostic Primer to Improve Detection
Source: Pathogens. 2020 Dec 12;9(12):1045. doi: 10.3390/pathogens9121045 (PMC7764570; doi:10.3390/pathogens9121045)
Supplement: Supplementary file 1 [file pathogens-09-01045-s001.zip › Suppl file S8- RT-PCR with BanMMV CP9-FV.pdf]

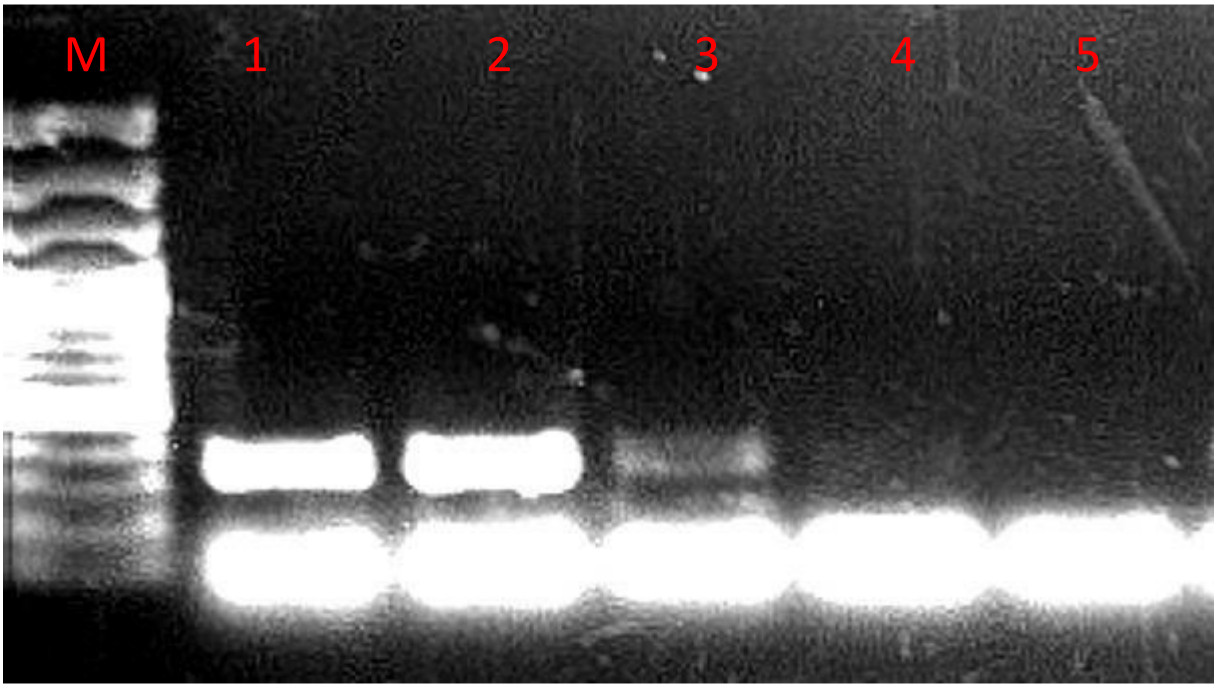

Fig. RT-PCR using BanMMV CP9 (and Poty1). M- Marker (100bp) ; Lanes 1 to 5 : ITC0763, ITC0763 (repetition), ITC1160, ITC1859 and healthy banana (negative control).
