# Supplementary material for: Identification of Divergent Isolates of Banana Mild Mosaic Virus and Development of a New Diagnostic Primer to Improve Detection
Source: Pathogens. 2020 Dec 12;9(12):1045. doi: 10.3390/pathogens9121045 (PMC7764570; doi:10.3390/pathogens9121045)
Supplement: Supplementary file 1 [file pathogens-09-01045-s001.zip › Suppl file S9- Photo gel-FV.docx]

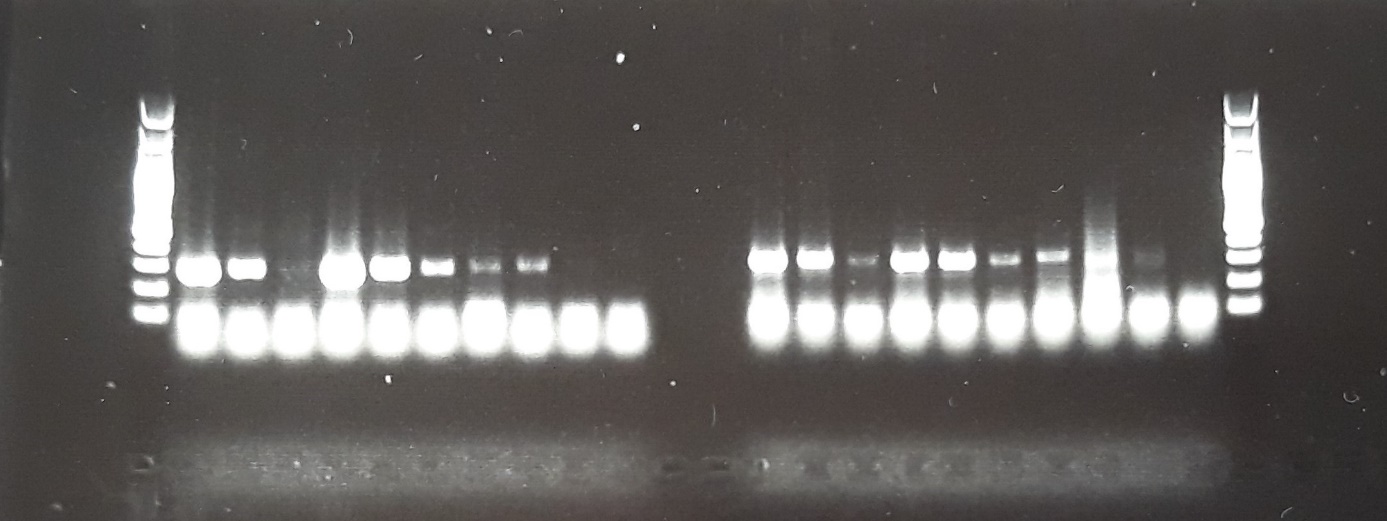


**280bp**pp

M

M

T-

T-

Sample 3

Sample 2

T-

**BanMMV CP2**

**BanMMV CP9**

Sample 1

Sample 1

Sample 3

Sample 1

Sample 2

Sample 1

With M : 100bp molecular weight marker and T- refers to negative control (healthy banana).

Sample1: ITC1859, Sample2: ITC1861, Sample3: ITC0412

The samples were tested in three contexts: sample not diluted, sample diluted 10x, and sample diluted 100x
