# Supplementary material for: Identification of Divergent Isolates of Banana Mild Mosaic Virus and Development of a New Diagnostic Primer to Improve Detection
Source: Pathogens. 2020 Dec 12;9(12):1045. doi: 10.3390/pathogens9121045 (PMC7764570; doi:10.3390/pathogens9121045)
Supplement: Supplementary file 11 [file pathogens-09-01045-s001.zip › Suppl file S5- Phylogenetic analysis-CP-FV.odt]

Figure. UPGMA phylogenetic trees inferred from CP nucleotide sequences of BanMMV and isolates from this study. The cyan dot denotes the single complete genome of the virus (GenBank accession NC_002729). The purple triangles denote the BanMMV isolates characterized during this study.
